# Interprofessional Education for Whom? — Challenges and Lessons Learned from Its Implementation in Developed Countries and Their Application to Developing Countries: A Systematic Review

**DOI:** 10.1371/journal.pone.0096724

**Published:** 2014-05-08

**Authors:** Bruno F. Sunguya, Woranich Hinthong, Masamine Jimba, Junko Yasuoka

**Affiliations:** 1 Department of Community and Global Health, Graduate School of Medicine, The University of Tokyo, Tokyo, Japan; 2 Department of Social and Environmental Medicine, Faculty of Tropical Medicine, Mahidol University, Bangkok, Thailand; University of Washington, United States of America

## Abstract

**Background:**

Evidence is available on the potential efficacy of interprofessional education (IPE) to foster interprofessional cooperation, improve professional satisfaction, and improve patient care. While the intention of the World Health Organization (WHO) is to implement IPE in all countries, evidence comes from developed countries about its efficiency, challenges, and barriers to planning and implementing IPE. We therefore conducted this review to examine challenges of implementing IPE to suggest possible pathways to overcome the anticipated challenges in developing countries.

**Methods:**

We searched for literatures on IPE in PubMed/MEDLINE, CINAHL, PsycINFO, and ERIC databases. We examined challenges or barriers and initiatives to overcome them so as to suggest methods to solve the anticipated challenges in developing countries. We could not conduct a meta-analysis because of the qualitative nature of the research question and the data; instead we conducted a meta-narrative of evidence.

**Results:**

A total of 40 out of 2,146 articles were eligible for analyses in the current review. Only two articles were available from developing countries. Despite the known benefits of IPE, a total of ten challenges or barriers were common based on the retrieved evidence. They included curriculum, leadership, resources, stereotypes and attitudes, variety of students, IPE concept, teaching, enthusiasm, professional jargons, and accreditation. Out of ten, three had already been reported in developing countries: IPE curriculum, resource limitations, and stereotypes.

**Conclusion:**

This study found ten important challenges on implementing IPE. They are curriculum, leadership, resources, stereotypes, students' diversity, IPE concept, teaching, enthusiasm, professional jargons, and accreditation. Although only three of them are already experienced in developing countries, the remaining seven are potentially important for developing countries, too. By knowing these challenges and barriers in advance, those who implement IPE programs in developing countries will be much more prepared, and can enhance the program's potential success.

## Background

Interprofessional education (IPE) is an effective tool to develop collaborations and efficiency among health workers of different professions. Interprofessional education (IPE) *occurs when students from two or more professions learn about, from and with each other to enable effective collaboration and improve health outcomes* [1]. Evidence reported in Cochrane reviews on the effectiveness of IPE has shown improvement of professional practice and health care outcomes [2], [3]. Moreover, IPE has the potential to improve future health workers' clinical and medical knowledge and clinical skills [2]–[6]. For medical professionals, IPE can help to reduce clinical errors in patient management [4], [7]. In this way, IPE can help to improve patient [3] and health worker [8] satisfaction. When individuals of different professions learn together, the experience can break down the professional wall between them, change their attitudes, and reduce stereotypes between professions within the medical field [9], [10]. Among students, IPE has been a useful strategy to help change their attitudes, develop their interests in patient care, and improve their medical and clinical knowledge [11]–[13]. Management of patients from different point of views (different professions) is important for the quality of care and wellbeing of the patients.

The World Health Organization (WHO) has endorsed IPE in light of its effectiveness [1]. In its guidelines for transformative medical education, WHO is calling on nations to foster IPE and integrate it in their existing curriculum to yield its desired effects. Integrating IPE in the traditional curriculum is a corner stone for its sustainability and cost effectiveness [14], which can help IPE adoption even in resource-constrained countries. However, due to limited evidence from developing countries, these guidelines were based on evidence derived from developed countries.

A paradigm shift in the epidemiological transition in lower and middle-income countries necessitates a number of health workers from various disciplines to work together to address the pertinent global health challenges. In such countries, the burdens of road injury and non-communicable diseases, such as diabetes, stroke, and cancer, are on the increase [15], [16]. Management of such patients requires a team of health workers to work together in collaborative ways [17]. For example, a stroke patient would need a paramedic, a physician, a nurse, a psychologist, and a physiologist for his/her better quality of care and life. An increase in such non-communicable diseases does not mean that the threats of communicable diseases are over [18]. The two will continue to affects millions in developing countries, especially in sub-Saharan Africa, where a chronic problem of human resources for health (HRH) remains a challenge.

IPE has the potential to reduce the HRH crisis [19] in developing countries if properly conducted. It may simplify task shifting when health professionals acquire the necessary competencies [20]. When medical doctors and nurses are trained together, they can acquire some of the skills of the other. Such skills and knowledge transfer can enable one to perform some of the tasks of another. In addition, IPE's role in interprofessional collaboration (IPC) can complement this process. In this case, the burden of patient care may be shared among available health workers as a team in the context of a health worker shortage. IPE may also help to ease the problem of poor HRH retention especially in hard to reach areas caused by the lack of incentives, motivation, and interest of health workers [21]–[23]. To this end, IPE and later IPC may help retain health workers because working in a team can help reduce the burden on individuals and increase their motivation towards their clinical work. The positive results of such collaborations may further foster the spirit of teamwork.

For many years, IPE has been conducted mostly in developed countries [2], [6], which provide most of the current evidence. Lessons learned through IPE practice have helped to shape and improve such programs. In contrast, limited evidence is available from developing countries [1]. Lack of evidence on IPE will necessitate rolling out IPE in developing countries based on the assumptions and tools derived from developed countries. This process may be more successful if it also considers the barriers and challenges encountered when implementing similar programs in developed countries.

In developed countries, IPE has had a number of challenges and barriers at its various stages including planning, initiation, and implementation. Although evidence is available for these challenges in developed countries they may be insufficient to extensively examine barriers and challenges in developing countries. However, lessons learned from other IPE programs are vital for implementing IPE globally and encouraging IPE programs in the developing world. This review aimed to 1) examine challenges and barriers to IPE, 2) collect lessons learned while implementing IPE, and 3) make suggestions as to what to expect when planning, initiating and implementing IPE in developing countries.

## Methods

We conducted this systematic review to examine challenges encountered while planning, initiating, and implementing IPE in various settings. Furthermore, we aimed to use evidence from well-conducted IPE programs to suggest efficient approaches to implementing IPE in developing countries, which are also suffering from a burden of HRH crisis.

In this review, IPE was the intervention of interest. The population of interest included students, staff, and faculty of medical, biomedical, and nursing schools and institution leaders or managers. We examined challenges or barriers encountered during IPE implementation and possible strategies used to overcome such challenges. The lessons learned from such IPE studies were used to make suggestions for IPE planning, initiation, and implementation in developing countries.

The protocol of this review was registered in October 16, 2013, at the PROSPERO database: http://www.crd.york.ac.uk/PROSPERO/DisplayPDF.php?ID=CRD42013006028. The protocol registration number is CRD 42013006028.

Based on the objectives of this review, we selected only studies that evaluated an IPE program or intervention. The inclusion criteria were studies concerning planning, initiation, or implementation of IPE. We included primary research conducted as cross-sectional quantitative and/or qualitative, prospective cohorts design, and retrospective evaluations of IPE programs. We excluded studies that did not have clear information on IPE planning or implementation. Other excluded articles included opinion articles, commentaries, editorials, reviews, and others that were not based on primary data. Studies on interprofessional learning outside of the health professions were also excluded.

Before the protocol development, we searched for similar studies, protocols, and ongoing reviews on a similar topic. Two reviewers (BFS, HW) conducted the search in the Cochrane Database of Systematic Reviews (CDSR), Database for Abstracts of Reviews of Effects (DARE), and Education Resources Information Center (ERIC).

Two independent reviewers (BFS, HW) conducted the search of evidence in selected medical databases. The included databases were PubMed/MEDLINE, CINAHL, PsycINFO, and ERIC. We limited the search to the articles written in English, with abstracts, and published within the past 25 years (November 1988-November 2013). We constructed the Boolean terms to capture studies on IPE that fulfilled our selection criteria for PubMed/MEDLINE. We used similar text words and MeSH terms as in PubMed/Medline for other databases.

**Figure 1:**
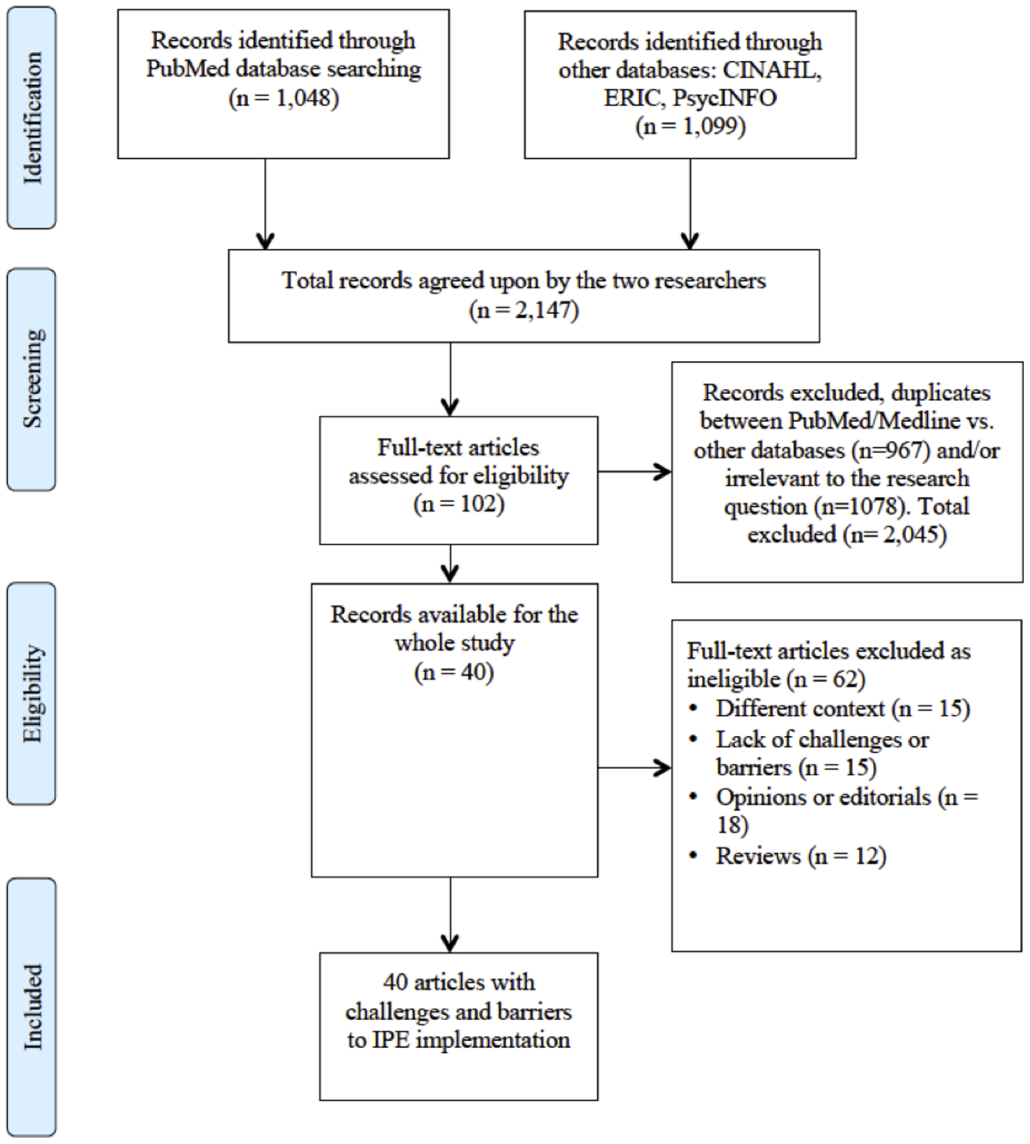
Diagram of information flow through phases of systematic review.

We retrieved a total of 1,048 articles from PubMed/MEDLINE, 879 from CINAHL, 11 from PsycINFO, and 209 from ERIC. Although we conducted a hand search of the Journal of Interprofessional Care, articles retrieved were similar to those found in PubMed. Therefore, a total of 2,147 articles were available for preliminary screening. We conducted the preliminary screening and excluded a total of 2,045 articles. Such articles did not meet our selection criteria based on content (n = 1080), or were duplicates between PubMed/Medline and other databases (n = 967). A total of 102 were thus available for in-depth screening. At this stage we further excluded a total of 62 articles due to lack of challenges of IPE (15), different context such as IPC (15), opinions or editorials (18), and IPE reviews (12). The remaining 40 articles were analyzed for this study. (Figure 1)

Table 1 shows the summary characteristics of the 40 selected articles on IPE programs or interventions. Because of the wide differences in study design, populations, settings, and presented results, it was impossible to conduct a meta-analysis. Furthermore, the outcomes of interest, barriers and challenges, are mostly measured qualitatively. Therefore, we opted to conduct a meta-narrative of the data collected. As we had only two articles for IPE programs in developing countries, we first reviewed the 38 articles from developed countries. Then after identifying their common challenges and barriers, we compared them to the two articles from developing countries to develop suggestions for IPE in developing countries.

**Table 1:** Characteristics of the selected studies, challenges, barriers, and efforts to address them.

	Country	Citation	Place of IPE conduct	Challenges/Barriers	Efforts to address challenges	Results/success
1	US	Brashers V, 2012	School of Nursing (SON) and School of Medicine (SOM), University of Virginia	Integrating IPE into curriculum of both schools; Learning level differences; Funding/resources; Institutional culture	A team from both schools was formed. It was comprised of key administrators, faculty, students, health system clinicians, chief medical officers, staff nurses, and nurse practitioners, committed to design and implement systematic IPE efforts; Curriculum: Comprehensive curriculum review by both schools to enhance existing courses and identify where new IPE experiences are needed for IPE core competencies; Learning levels: Committee explored IPE in both curricular and extracurricular learning to provide new and clinically relevant IPE experiences across the learning continuum; Funding: schools integrated IPE in their routine activities; a grant was sought for the expanding projects. A funding deal was made to seek external grants and identify key sources; Institutional culture: The progress of IPE changed such culture of SOM and SON and the health system, resulting into more interest and commitment to IPE and IPC	Sustained IPE with more committed parts; Faculty members developed new IPE experiences; Comprehensive program for evaluation is being developed on students' IPE competencies; Two external and several internal grants have been awarded for sustainable IPE; Students' knowledge on roles and team work competencies increased IPE teams from various fields; Students' participation increased in community and internationally Collaborative research increased
2	UK	Fook J, 2013	Faculty of Health and Social Care Sciences (FHSCS) made of three universities in the UK	Funding/resources; Different conceptualizations of IPE (top-down vs. student-centered IPE); Contrasting systems and teaching processes; Staff relationships/tensions; Lack of central planning	Resources: Staff took on additional responsibilities and in some cases they were deployed from other activities. Outcomes of the program were a driving factor for the program; Acceptance of the IPE was a positive element of the workplace culture. Vast majority of staff signed on for the program; Despite the lack of central planning, the institutions had charismatic leaders in terms of vision and contribution; Students provided feedback, commitment and strong belief in its importance	Wide ownership of values across the academic community and strong commitments brought about sustainability and development of this IPE in the UK
3	Singapore	Jacobs JL, 2013	Four academic units at the National University of Singapore: Center for Nursing Studies; School of Medicine; Department of Pharmacy; and Faculty of Dentistry.	Curriculum: No underlying principles of competency framework; Schedule: Coordinating students and teachers from different units; Leadership: No centralized group charged with and empowered to sustain IPE	Curriculum: Adopted an international IPE competence framework identified by the committee and designed as a sustainable IPE platform based on six competencies: team work, roles/responsibilities, communication, learning, patient focus, and ethics; Schedule and curricula: Interprofessional Core Curricula (ICC) was recognized where learning opportunities from each unit that were part of core curricula become part of the ICC. Also Interprofessional Enrichment Activities (IEA) was developed, where unique, incentivized activities were recognized. A trainee must engage in more than one academic unit (two or more competencies) and be assessed; Student and faculty development through informal and formal meetings before the start of the program; Evaluation made an integral part of the program	Lessons: Adding new programme requirement to an academic programme is demanding; Student feedback is essential; and recognition of staff's participation is important for success. Success: IPE was successfully started and the university is planning a centralization; and could be a model replicable to other universities
4	Canada	Steinert Y, 2005	McGill University; 16 Canadian faculties of medicine; and departments of family medicine	Curriculum gridlock: complex timetable and limited resources; Differences in students' characteristics: age, learning styles, and motivation; Faculty attitudes: condescension and defensiveness, lack of respect, sense of academic elitism, and silo approach	Faculty development strategies: Having faculty from diverse disciplines come together for a faculty development session; promote dialogue and exchange; discuss professional roles, overlaps, and address causes of friction; finding common ground through teaching; faculty development strategy can help foster IPC and introduce a new collaborative culture	
5	UK	Anderson ES, 2009	Leicester model of IPE: A multi-disciplinary health and social care center, and Prince Philip House	Curriculum: Time constraints and timing of modules, medical dominance; Students' preparation for working in areas of poverty; Unfamiliarity with teaching different cadres and unpreparedness of faculty; Medical phrases and differences among professionals	They addressed challenges by formation of an IPE steering group consisting of staff from each discipline and other involved parties to receive and act on evaluations; Students' concerns on curricula: IPE integrated in all curricula Concern on working in poor areas: Students reminded at the beginning of the course; Curricula timing: Students placed at the center of the curricula. In the Leicester model, students designed their curricula; Medical phrases rewritten in appropriate terminologies for different disciplines; Financial partnership for the teaching budget	Steering group continued to tackle problems as they arose and lead into a sustainable IPE. Sustainability was also brought about through ensuring integration of education research in development process, and engaging participants and practitioners to lead into team work
6	Canada	Cameron A, 2009	Nine health sciences faculties of the University of Toronto	Schedule/timing; Content/style of sessions; Organizational issues; Needs for further knowledge/skills; Lack of enthusiasm- faculty missed attendance	No explanation	Planning smaller classes can foster interactions; Students in the beginning of the program are more enthusiastic; Involving students in planning process is important
7	Canada	Gilbert JV, 2005	University of British Columbia	People: attitudes and stereotyping of the faculty and students- within the university, academicians, and students; Professional associations are powerful and establish guides for practice within a profession; Specialization; Financial barriers- cost of curriculum time and associated cost; Require more staff; Financing: differential salaries, faculty budgets, and students’ fees; Cost of service, research; Accreditation demands; Academic demand vs. practice demand; Management	Solutions to such barriers were not offered directly, but recommendations were provided to address these challenges to ensure sustainability of IPE	
8	UK	Lindqvist SM, 2007	Nine different health professional student at the University of East Anglia	Schedule: Students thought it was time wasting; Demanding: Facilitators viewed having students from different fields as a complex activity and demanding; Different learning needs	IPE itself was a driving force: Students enjoyed the program, Smaller team groups: facilitators felt that it provided them with better knowledge of team work	IPE developed a better IPC for faculty
9	Canada	Salfi J, 2011	Nursing students in B.Sc.N, Canadian Interprofessional Health Collaborative (CIHC)	Curriculum design and integration: time consuming and costly; Lack of interest for faculty and students; Difficulty in securing clinical placements	Framework on IPE was developed with 4 levels: Level 1-IP competency, 2-Competency practice IPC, 3-IP competency-IPC actual practice, 4-Becoming effective on health care team. To facilitate the process of integration of IPE into professional curriculum, faculty members from each field were involved in developing, evaluating, and sustaining the initiative. Students and faculty were involved in special pre-session programs—dedicated IPE days—which helped to break walls dividing traditional professional programs. Credit for extracurricular activities: awarding credits to nursing students engaging in IPE improved participation, enthusiasm	Lessons: Creative curriculum tailored to the needs and characteristics is important; Eased the adoption of IPE framework into a curriculum by capitalizing on what already existed; Effective communication, respect, shared decision making, collaborative leadership, problem solving, and conflict solving are fundamental.
10	Egypt	Hosny S, 2013	Faculty of Medicine, Suez Canal University	Curriculum structure, course or module, complexity; Attitudinal barriers	Students' evaluation to maximize educational impact of the process and feedback from faculty; Address attitudinal barriers	Identification of champion of IPE, for stronger leadership; The impact of IPE felt
11	US	Pecukonis E, 2013	University of Maryland, IPE program	Confusion of students' supervisors' roles; Lack of clear expectations Time planning/curriculum; Emotions, conflicts between scholars; Diversity in age, group demographics	Clear structural hierarchy was made; Preparing clear road map of the course including topic selection, and students' expectations before the course, through 2-day retreat. Assisting students to go through the process and solve conflicts: self-reflection, awareness, and mindfulness. Learning which group benefits from the course more and helping them through their differences	Impact: MCH curriculum was integrated within the school. A significant increase in number of students expressing interest in career in MCH (68% increase in ten years)
12	US	Brennan CW, 2013	Five Veterans Affairs Medical Centers in the US	Tendency of each professional to overvalue own profession		Pre-course discussion on background, work experience, strengths and weaknesses. Embrace multiple perspectives
13	Canada	Church EA, 2010	Six rural Canadian communities	Curriculum did not meet needs of participants; Logistical difficulties: video conference	Strong relationships between facilitators and participants; Materials linked with professionals' experiences; Small group interactions; Continuity of facilitators; Spread over period of time	Covered 125 professionals. Benefited more professionals with mental health background, as intended
14	Finland	Juntunen A, 2004	Four polytechnics provided IPE for nurses, social workers, and physiotherapists on elderly care	Lack of adequate supervision/support; Lack of preparations by tutors; Limited knowledge and skills of teachers Time inadequacy	15 units of European credit module in elderly care was developed	150 students from nursing, social welfare, physiotherapy, and gerontology faculties registered, 112 completed the course. 25% students dropped out
15	Namibia	Wessels Q, 2013	School of Medicine and School of Pharmacy, University of Namibia	Resources: Budget and workforce; Leadership: to guide investment, lab, teaching, and service provision; Balance between needs and demands in public and private sectors	Opportunity to integrate the program into existing programmes: Alignment of learning environment, learning objectives, and teaching methods; Infrastructure developments: buildings, labs, and other teaching spaces encourage IPE; Institutional agreements in place: Outside university, faculty development, and visiting lecturers	Holistic approach needed to enhance teaching and system in its entirety: methods and assessment; Learning objectives should be aligned with the current social and health needs and directed under institutional leadership
16	Canada	Barker KK, 2005	Family health center at the University Health Network-Toronto	Professional stereotypes; Attitudinal barriers; Tensions between cadres; Mixed messages between trainers on collaboration		Trainees in IPC gained knowledge on roles and ultimately changed their attitudes, and behaviors; They reported professional growth
17	Canada	Reeves S, 2006	Teams involving social workers, community psychiatry nurses, psychiatrists, administrators	Poor attendance of medical staff; Lack of time for implementation: heavy work load; Lack of support from management; Perceptions of senior staff		Time is needed to conduct such workshops and IPE initiatives; Schedules should take into consideration of professional's workload; Involvement of administration/management is important
18	UK	Forte A, 2009	IPE scheme for allied health sciences at London South Bank University	Teaching style: lecturers exhibit a dominant style pertinent to their professional tradition, perceived as unsuitable for some students; Compiling case studies for students that normally do not work together is challenging	Combination of professions within teaching and student groups solved the second challenge; Authenticity was ensured using patient pathway approach in developing case materials	Identified opportunities: Development of effective communication between professional groups to break barriers and reduce stereotypes
19	UK	Carlisle C, 2004	Institutions based in North West region, England: Students, academic staff, practitioners, and patients	Organizational: rely on motivation of all involved; Course structure and curriculum; Choice of teaching methods; Lack of clarity on clear aims and learning goals	Problem based learning (PBL) was seen as the means of providing the right teaching environment for interactive learning and means of amalgamating different learning styles into the curriculum	
20	Norway	Clark PG, 2011	Clinical care settings in Oslo	Schedules/organization; Rigidity of curriculum; Faculty attitudes; Lack of perceived values	Potential facilitators of IPE included; Funding availability; Administration support; Flexibility in the curriculum	Bridging IPE-IPP gap requires educating leaders in both settings about the resources needed for teamwork, linking clinical-educational settings, and advocacy
21	UK	Courtenay M, 2013	Medical students at the university of Cambridge and non medical prescribing students at Anglia Ruskin university	Differences in knowledge between nurses and doctors in pharmacology; Professional jargons; Organizational structure: Schedule, location, and balance		
22	US	Tullmann DF, 2013	School of nursing and school of medicine	Loss of interest of one party; Lack of enough time	Despite lack of interest by school of medicine, the other parties continued with the existing plans; The driving forces were the loss of time already invested and the importance of the program	
23	US	MacDonnell CP, 2012	Warren Alpert School of Medicine, Brown University (AMS), College of Pharmacy, University of Rhode Island (URI)	Scheduling logistics of holding one day practicum with large number of students; Selection of appropriate level of study among students; Faculty and practitioners' attitude towards the program; Space and proximity of academic institution	Determine the appropriate level of education for the three student disciplines; Planning for faculty development to embrace the IPE program in the future	Developed the framework of an interprofessional education curriculum to be disseminated to administrators at AMS, URI and Rhode Island College
24	US	Headrick LA, 2012	Case Western Reserve University, John Hopkins University, Pennsylvania State University, the University of Colorado, the University of Missouri and the University of Texas Health Science Center	Schedule; Mismatch between students' ages and clinical experiences; Students' lack of knowledge about each other's backgrounds and strengths; Students' uncertainty about the importance of quality improvement and patient safety content; Faculty unfamiliarity with quality improvement and patient safety content; Creating meaningful clinical experiences in quality improvement and patient safety for more than a few students	Clear commitment from dean's offices and interprofessional faculty leaders; Having student teams schedule their own meeting time; Planning in advance, before other schedules are set; Seeking learning activities in which students with different prior experiences can be equally successful; Making differences apparent and using them to create learning experiences that take advantage of each group's strengths; Making time for students to get to know one another; Providing encounters with real patients; Including students on the educational planning team to help create attractive and energizing learning activities; Providing faculty development prior to and specifically for the educational intervention; Working with partner health care organizations to identify ways in which students can contribute to quality improvement and patient safety; Customizing the experience to the clinical site and sharing best practices across sites	
25	Norway	Aase I, 2012	Nursing schools	Logistical and organizational challenges; Competing demands		
26	US	Djukic M, 2012	College of Nursing, New York University and Bouvé College of Health Sciences, Northeastern University	Complex educational infrastructure; Lack of physical space proximity and availability; Limited faculty resources needed to deliver IPE to a large number of students	Using asynchronous, modular, Web-based learning that can be integrated into the existing curricula	The curricula products are available for public use and can be accessed online
27	Malaysia, Philippines, Korea and Japan	Lee B, 2012	Medical schools in Western Pacific Region countries	Rigid curriculum; Lack of financial resources; Schedule/calendar; Lack of administrative support; Lack of reward for faculty; Lack of perceived value; Turf battles; Faculty attitudes; Student acceptance; Classroom size		Promoting the dissemination of IPE initiatives in the region is needed
28	USA	Jones KM, 2012	Colleges and schools of Pharmacy	Lack of appropriate facilities; Lack of personnel resources; Lack of financial resources; Not a priority at the time		The study did not implement any solutions but raised possibilities including: Providing electronic resources such as cases or simulations; Providing standardized assessment tools; Providing online resources for faculty training; Facilitating partnering
29	Australia, New Zealand	Lapkin S, 2012	Universities in Australia and New Zealand that offer nursing, pharmacy or medical programs	Timetable restrictions; Lack of appropriate teaching and learning resources; Funding limitations		Some recommendations arose from the study to benefit the IPE: Academic staff development; To avoid medication errors, teamwork and interprofessional cooperation should be taught through IPE experiences
30	New Zealand	McKimm J, 2010		Determining the right stage of readiness for students to engage in IPE; Number of students; Timetable constraints; Differences in experiences; Commit to invest in IPE; Recruiting, training and supporting expert facilitators and IPE ‘champions’; Stereotypes, attitudes and professional identity; Professional jargons	Student's evaluation to maximize educational impact of the process, and feedback from faculty; Address attitudinal barriers	It is important to identify champions of IPE for a stronger leadership; The impact of IPE in this university was felt Training in IPE was provided to faculty, clinicians to incorporate it into training and activities
31	US	Aston SJ, 2012	Western University of Health Sciences, Thomas Jefferson University and Rosalind Franklin University of Medicine and Science	Curricular; Faculty participation; Logistics: location and resources; Student workload; Lack of accrediting bodies	Western U developed 300 faculty and qualified external participants to facilitate IPE; Embraced an educational model based on eight central tenets of education: Interprofessional learning, student-centered learning, student ownership in the learning process, faculty as facilitator or mentor, integration of adaptive curriculum, competency-based instruction, assessment-validated change, and evidence based best educational practice. All of these are heavily embedded in the mandatory IPE courses; Training of 40 mentors from the eight professions. Outside experts were recruited to help faculty with small-group facilitation	Western U developed and continues to refine a three-phase program. I-case based, II-experiential teamwork and III-clinical care portion. An innovative inter-institutional IPE program was created with Oregon State University and Linn-Benton Community College
32	Hungary	Kobor K, 2009	Szechenyi Istvan University, United Institute in Health and Social Care	Hierarchy between different sectors and within the sectors; Academic accreditation processes; Lack of driving force in local and national governments and public administration	Collected information on good practices in IPE, translated key texts into Hungarian and produced a Hungarian brochure about IPE; Ran workshop on IPE for service managers, practice teachers and lecturers Developed IPE network in Hungary; Developed new IPE courses at different educational levels	The Department of Applied Social Sciences at the University of Debrecen also now offers a Social Health Worker MSc course for the development of IPE, popular in Hungary
33	Canada	Ho K, 2008	Universities of British Columbia, Alberta, Ottawa, Dalhousie and Memorial University	Organizational structures Funding allocation by faculty; Schedules Conflicts between professional practices and between academia and professions; Faculty attitudes	Built relationships that fostered collaboration and a willingness by all involved to demonstrate flexibility and compromise in developing programs; Started the program with champions (including deans, associate deans and directors)	
34	US	Liston BW, 2011	Medical students in internal medicine rotation	Scheduling alignment; Time in the existing curriculum; Resources in time and money; Medical student interest and beliefs in the value of IPE; Faculty attitudes		
35	US	Blue AV, 2010	Medical schools	Funding limitations; Lack of classroom space; Lack of clinical space; Academic calendars and schedule; Lack of comparable readiness of students		
36	US	Smith KM, 2009	College of Pharmacy	Professional culture; Scheduling challenges; Curricular concerns; Limited resources; Lack of conceptual support; Insufficient classroom space; Differences in baseline knowledge of students; Defining nature of disciplines and their innate differences; Lack of infrastructure to reward faculty members for engaging in IPE approaches; Lack of consistent focus on IPE among accrediting bodies in the academic healthcare sector		Recommendations: Change to an IPE focus must be consistently supported and stimulated by the accreditation standards for all healthcare professions; Academic incentives for units, as well as individual faculty members, to pursue IPE initiatives; Examination of each discipline's curricula to identify core knowledge and skills required for successful graduates; View IPE as a continuum with small forays at the onset; Engaging instructors from other units or degree programs in educational delivery
37	Canada	Hoffman SJ, 2008	NaHSSA's Third Annual Conference	Lack of funding; Lack of IPE clinical placement; Curricular challenges; Lack of institutional and/or administrative support; Lack of student interest in leading IPE activities; Lack of IPE research opportunities; Lack of faculty mentorship and/or guidance		
38	UK	Priest HM, 2008		Logistical challenges; Different professional experience and different levels of experience; Inherent tribalism and tendency for participants to gravitate towards their own professional group	Planned the sessions well in advance; The facilitators met regularly to plan the sessions and consider how to aid students' learning across sites.	
39	US	Rafter ME, 2006	Dental schools	Lack of time in the curricula of the health profession schools; Long physical proximity between the different schools; Lack of administrative and faculty support for IPE; No incentives either in terms of finances, promotion or career development; Financial limitations; Lack of scientific evidence for the effectiveness of IPE		
40	UK	Stew G, 2005		Lack of commitment, motivation, and assistance from academic staff to students; Clinician-led sessions: Reinforcement of hierarchy divisions within multidisciplinary team; Tutor-led sessions: Control of IPE by academic staff may disempower clinicians. The students may not value the activities if the lecturer seems out of touch with clinical reality	Recognizing and rewarding student effort through the award of credit within their curricula and recognizing qualified staff involvement as formal continuing professional development; Involving students in selection and presentation of session contents and relating them to their own placement learning objectives; Involving practical session's educators in preparing and delivering the sessions and enhancing the clinical credibility of academic staff	

We adhered to the Preferred Reporting Items for Systematic Reviews and Meta-Analyses (PRISMA) [24], to conduct this review and report its findings (Checklist S1).

## Results

### General description of the included studies

A total of 40 studies were eligible for analysis out of 2,147 studies retrieved. Only two studies reported findings from developing countries, which were Egypt [25] and Namibia [26]. Two studies reported findings from more than one country [27], [28]. A total of 13 studies were conducted in the US [14], [29]–[40], indicating a high number of IPE programs conducted there. Eight studies [41]–[48] were conducted in the UK; nine studies [13], [49]–[56] were conducted in Canada; two in New Zealand [28], [57] and Norway [58], [59]. Among the selected studies, Australia had one study [28] and so did Singapore [60], Finland [61], Hungary [62], Egypt [25], and Namibia [26]. IPE was conducted in medical universities including teaching hospitals in 30 studies and in other health and academic institutions in eight studies. Because of the nature of our research question, most of the selected studies focused on IPE program evaluation, some in longitudinal designs, and the majority in qualitative design. All the selected studies explained or mentioned the challenges encountered while initiating or conducting IPE. However, a total of 16 studies offered no solutions to such challenges or means used to address them.

Challenges to establish and/or implement IPE varied among institutions and programs. Table 1 shows that common challenges and barriers revolved around common themes. They included IPE curriculum, leadership, resources, stereotypes and attitudes, variety of students, IPE concept, teaching, enthusiasm, professional jargons, and accreditation.

### Challenges and barriers in implementing IPE in developed countries

#### 1) Curriculum

A total of 30 studies reflected IPE curriculum as an important challenge to implementing the program. Because of differences in the curricula, curriculum challenges in this research were divided into curriculum content, integration, time and schedule, and course rigidity.

IPE course contents, structure, and style of lessons were mentioned as challenges in 10 studies [13], [34], [38], [39], [45], [51], [52], [56], [57], [61]. As for a solution to this, problem based learning (PBL), when it was tried, improved the IPE course because it was based on cases pertinent to the area of the institution involved [45]. It helped to increase interest and attracted more students. Others solved their challenges through a stepwise course structure that started with improving knowledge competence of the lowest level, followed by more complex practices in subsequent levels until IPC was reached in the highest level [51]. Students' evaluation of course content and faculty evaluation of and feedback to students also helped to improve course structure [57]. Innovative approaches including web-based IPE courses also could provide a solution in some settings [34]

The lack of integration of the IPE curriculum into the traditional academic frameworks of the professions involved was an important barrier in one study [51]. As a solution, the program involved the institution's faculty members from the relevant fields to develop, evaluate, and sustain the initiative.

Time and scheduling opportunities to implement IPE were the most common curriculum challenges found in 15 studies [13], [27], [29], [33], [35], [37], [42], [43], [46], [49], [54], [55], [58], [60], [61]. To solve this barrier, some implementers made the IPE subjects and other learning activities a part of the existing core curriculum of the involved professions [60]. Those learning activities were incentivized though grading points [60], [61]. Involving professionals [29], [36], [47], [49] and students [42] in the early stages of IPE curriculum preparation alongside their professional curriculum will help the students plan a flexible curriculum [55], [58]. It will also help to commit the leadership [33] into addressing some of the time and scheduling barriers.

Two studies pointed to the rigidity of the IPE course structure as a barrier to its sustainability [27], [61]. A flexible curriculum aligned with the existing individual school's curriculum may attract and keep students in the program. It may also give time to professionals to participate in other activities while engaging in IPE. Students could participate in the IPE program without altering their professional schedules.

#### 2) Leadership

Poor planning [41], lack of coordination and organization [13], [40], [45], [55], and lack of interest or support by administrators [27], [32], [47], [54], [56], [60], [62] were some of the leadership challenges encountered while initiating and implementing IPE. To address leadership challenges, IPE teams should identify committed champions to spearhead their program [41], [45]. Institutional agreements, if formalized, can sustain IPE even when such IPE champions are no longer involved. Committees formed by the IPE actors should get involved from the planning stage [32], [55], including seeking support, especially financial support, from outside their institutions.

#### 3) Resources

Lack of funding or uncertainty thereof can affect initiation and implementation of IPE [14], [27], [28], [34]–[41], [47], [50], [55], [56], [59]. Funding is needed for curriculum development, payment of costs and remuneration of staff, staff training to be competent in managing cadres of different disciplines, research costs, and costs for running the program [50]. Additional costs are involved for students' tuition fees and studying materials. Unless the university incurs such costs, running the IPE program cannot be sustainable. IPE programs could thrive using various strategies. Some institutions integrated IPE into their mainstream curriculum to reduce the costs of running parallel programs [14], [37]. This also helped to increase IPE sustainability because it was aligned with the same learning environment, teaching objectives, and methods that already existed in their institutions [36]. In addition, this helped to reduce the need to recruit new faculty, which provided additional savings [41], [55]. Encouraging results in implementation of IPE programs were mentioned as a driving force for staff regardless of the burden of work [41].

IPE is usually conducted in existing classrooms. These classrooms can be small for IPE classes, which usually include more students than the classrooms were designed to hold. Also, IPE can include students from multiple institutions, which may not be in close proximity to one another. Lack of physical space [34] and longer distances to such classrooms or appropriate facilities for clinical practice [34], [35], [38] were cited as important challenges to conducting IPE programs. To solve these challenges, and where technology allows, a web-designed IPE could be used to deliver such classes [34]. The modules could be integrated into the parallel curriculum. Also, smaller numbers of students in IPE classes could help to improve interactions [52] and reduce the need for larger classrooms.

#### 4) Stereotypes and attitudes

Attitudes [27], [36], [49], [53], [55] and stereotypes [13], [53] held by faculty members are barriers to IPE. Preferences of trainers towards their own professions can undermine the learning process for students who belong to other professions. The medical profession is usually perceived as dominant to other professions [49]. As a consequence, medical doctors tend to over-value their cadres [30]. In most cases, other professions wait for medical doctors to make decisions or lead. This is not healthy for interprofessional teamwork. It creates classes among professionals and impedes collaboration and teamwork to implement IPE. Also, the more the professionals are specialized, the more difficult it is for them to collaborate [13]. Therefore, they may leave IPE for low, basic, or elementary education. Such professional stereotypical attitudes can be transferred to their students [35] and complicate IPE through creating emotions [29], tensions [53], and conflicts [29], [41] among faculties and students.

How might such faculty attitudes towards IPE be overcome? In some programs, shared values for IPE and its expected results provided a strong drive to overcome attitudinal issues [41], [58]. Faculty development of strategies before implementing IPE can minimize the effects of professional stereotypes [36], [49]. It can also increase the sense that IPE is important for teamwork and collaboration among different cadres. Others proposed solutions include the identification of stereotypes and attitudes by staff themselves [29] or through evaluation by the students.

Similar to their teachers, stereotyping is a common problem among students of different cadres [13]. The health professions are based on different disciplines, values, and philosophies. Students pick up such attitudes from their teachers. They can therefore display such attitudes towards other cadres and cause problems in running IPE programs.

Institutional, administrative, or professional bodies' attitudes: Institutional culture [14] and lack of interest towards IPE can make it difficult to implement such programs. Inherent professional tribalism [39], [47] and preference towards one's own traditional practice by one cadre over another [55] can cause the left out cadre to feel unwelcome or devalued in the program. Professional organizations that are constituted entirely of members of one cadre also have been barriers to IPE [49]. The progress of IPE and the positive results thereof may help to reduce such institutional attitudes and increase interest in IPE programs [14]. Involving professional bodies from inception and explaining the role of IPE in their own professions can break the gridlock among such bodies and increase their support towards implementation of IPE. If the champions of the program are school administrators such as deans and faculty leaders, these institutional barriers can be more easily avoided [55].

#### 5) Variety of students

Students of different professions have different characteristics [49]. They may have different learning needs and basic knowledge levels. For example, nurses and doctors may have different approaches to patient care. Combining such students in a common course without acknowledging their differences [33] could be a barrier to implementing the course [14], [46], [47], [57]. Diversity of students, if unaddressed before the course, may lead to confusion in IPE and in their regular professional courses [29], [33]. Successful IPE models acknowledged differences among professions in advance. Students then could learn about each profession and were taught to expect differences of opinion. Conflicts that arose were dealt with immediately and used as lessons to prevent future conflicts. Students' learning needs and capacities were assessed before the course, and common characteristics among different professions were used to plan how to conduct a successful IPE that could benefit all students. Methods such as problem-based learning and cases that were pertinent to all professions involved were employed.

#### 6) IPE concept

The concept and methodology of IPE programs differ from one institution to another [41]. Some consider a top-down approach the ideal model, where IPE is planned and implemented solely by the administrators. Others consider a student-centered model as ideal, where students plan their own IPE programs. Although IPE is effective in improving IPC and professional relationships, the effectiveness of one system over the other remains unknown. With the top-down approach, students are left on the receiving end. They are not part of the design, scheduling, or implementation of IPE. Ownership and enthusiasm may thus be reduced. The IPE concept also is not standardized in most programs. In most cases, by simply studying together the course was considered IPE, even without a clear curriculum. In such situations, the sustainability and longevity of the program remains doubtful. Professions still question the effectiveness of IPE [40]. Because of the lack of clarity and strong curriculum, students remain skeptical of IPE [59]. They might not have clear expectations [29], [36] because the IPE course did not have clear aims and goals from the beginning [38], [45]. To help solve this challenge, a clear curriculum should be prepared, involving students and other stakeholders throughout the process. The course should be aligned with the school calendar, and goals and expectations should be explained to all participants before starting the course. Stakeholders should own the course and regular feedback and evaluation should be performed. The course should be flexibly modified based on evaluation results.

#### 7) Teaching

Faculty encountered several challenges while teaching IPE. IPE classes were sometimes unusually large compared to regular classes, making it harder for faculty members to interact with students [59]. Because of the differences among cadres, they had to adopt new teaching styles [44], [45]. For example, some faculty members might not have been experienced in PBL but had to use it. Moreover, each faculty member might give a different message or professional opinion [53]. This can complicate teaching and consensus might take longer to reach. Differences in professions also can mean differences in learning needs and levels of understanding on various topics [43], [44]. Lack of existing research or resources to conduct original research can also impair teaching [56]. Insufficient skills, knowledge and competence of faculty members can hinder the teaching of multiple professions [50], [57]. Lack of preparation before the program, including faculty training, can contribute to insufficient competence [57]. Contrasting systems [41], differences in and unfamiliarity with teaching methods and styles for other cadres [33], [42], and lack of background information regarding the students involved [33], can also challenge IPE implementation.

#### 8) Enthusiasm

Lack of enthusiasm can result from a poorly planned IPE program. When a top-down approach is taken, participants may not be involved with developing the curriculum and planning the schedule. They also may not be informed about the importance of the program. Then, both students [57] and teachers [13], [27], [51] may end up with frustration and lack of interest. This can result in poor attendance [54], dropouts, and natural death of the program. In some instances, administrators [62] and one school [31] lost interest and lost the driving force behind IPE because they did not believe there would be a positive outcome [38].

In other instances, efforts were made to boost the enthusiasm and interest of parties involved in IPE. Such efforts included offering students rewards such as credits for attending IPE [51], including the IPE course in the mainstream curriculum [51], and expanding the practical base by using PBL. Even when other aspects lose focus, IPE can still yield its intended results if a strong team is in place [31]. Commitment from administrators, especially deans, is vital to IPE operations. Including such leaders in the program can boost enthusiasm [33]. Training and retraining facilitators and staff can also improve their involvements and enthusiasm [62].

#### 9) Professional jargons

The use of specific terminologies, especially scientific and medical terminologies, acronyms, and pharmacological names, were mentioned as a challenge in implementing IPE [42], [46], [57]. Changing such terms is unlikely, but explaining them when they are used for the first time can help ameliorate such a challenge. Familiarizing students and professionals with the use of such terms in advance and during the coursework, and providing printed documents of special terminology deemed difficult is also important.

#### 10) Accreditation

To standardize IPE, it is important to accredit the course. There are no specific bodies or institutions to accredit such courses yet [36], [39]. This makes IPE seem to be just an additional requirement rather than a serious course for participants [62]. Accreditation of IPE can also help to structure the curriculum in a standardized manner [36]. Despite the lack of such bodies and an accreditation process, institutes adopted IPE models that were successful in other regions [36].

### Relevance of the 10 challenges in implementing IPE to developing countries

Table 2 shows how the above 10 challenges and barriers in developed countries are included in the literature from developing countries. In this review, only two studies were available from developing countries. These studies also described a number of challenges in implementing IPE. For example, an IPE program in Egypt [25] found that IPE curriculum structure and course or modular complexity were important challenges. Moreover, attitudinal barriers made it difficult to implement the IPE program. In Namibia [26], the IPE program faced resource constraints that included budget and workforce. The need to have strong leadership to guide investment, teaching, and service provision was a necessary ingredient to improve IPE program performance. In these two examples, integration of the IPE program in the mainstream professional curriculum was mentioned as a cornerstone towards building a strong and sustainable IPE program. The three challenges described in developing countries were also common in IPE programs conducted in developed countries. Other challenges extracted from examples of developed countries therefore may be considered potentially relevant to developing countries as well.

**Table 2:** Challenges to implementing IPE in developed and developing countries and suggested solutions.

No	Challenges to implement IPE	Developed countries	Developing countries	Suggested solutions
1	IPE curriculum challenges: content, curriculum integration, time and schedule, rigidity	[13], [27], [29], [33]–[35], [37]–[39], [42], [43], [45], [46], [49], [51], [52], [54]–[58], [60], [61]	[25]	Involve students and faculty in early stages of curriculum development [29], [36], [45], [47], [49]; Students and faculty should evaluate course contents provide feedback of the IPE [51]; Integrate IPE into the existing core course curriculum [60]; Commit the leadership in addressing time and scheduling barriers [33]; Use innovative approaches using web-based IPE wherever possible [34]
2	Leadership weakness	[13], [27], [32], [40], [45], [47], [54]–[56], [60], [62]	None	Identify and use committed champions on IPE to spearhead the program [41], [45]; Put in place institutional agreements to help sustainability; Involve the IPE committee from the planning phases [32], [55]
3	Resources: financial challenges, physical infrastructure and distance	[14], [27], [28], [34]–[41], [50], [55], [56], [59]	[26]	Integrate IPE into the mainstream curriculum to save operational costs by using available resources and infrastructures [14], [37]; Build on positive results to encourage funding, institutional support, and other resources [41]; Conduct IPE on existing infrastructure using the same faculties; Use web-designed IPE to help solve distance and infrastructure barriers whenever possible [34]; Conduct small IPE classes to help minimize the need for larger classrooms and to improve interactions [52]
4	Attitudes and stereotypes	[13], [14], [27], [36], [39], [47], [49], [53], [55], [58]	[25]	Implement faculty development programs before the starting IPE [36], [49]; Help faculty to identify stereotypes by themselves [29]; Involve professional bodies from the inception of IPE and explain the role it can have to break gridlocks among them; Involve deans and faculty leaders to break institutional barriers [56]
5	Students' characteristics	[14], [29], [33], [46], [47], [49], [57]	None	Acknowledge the diversity and learning needs of each group before starting IPE; Use PBL to help stimulate learning by different professions
6	IPE concept	[29], [36], [38], [40], [41], [45], [59]	None	Set a clear curriculum and involve all parties before the beginning of and throughout IPE process; Align IPE courses within the school calendar and explain goals before taking initial steps; Provide regular feedback and evaluation to help clear misconceptions
7	Teaching barriers	[33], [41]–[45], [50], [53], [56], [57], [59]	None	Encourage faculty to set adequate time for preparations to improve their competency [57]; Provide training to staff on IPE training methods [42], [57], [67]; Provide the staff with adequate information on students involved including their background and special learning needs [50], [57]
8	Enthusiasm	[13], [27], [31], [38], [51], [54], [57], [62]	None	Incentivize students who attend IPE courses through grade points or credits to boost participation [51]; Expand practical base using PBL; Involve institutional leaders such as deans to boost enthusiasm [33]; Train and re-train staff and faculties to improve their involvement [62]
9	Profession jargons	[42], [46], [47], [57]	None	Familiarize students with professional terms before the beginning of a class/session; Provide students with printed materials to refer in case the terminology deems difficult but important
10	Accreditation	[36], [39], [62]	None	Set country's or regional's accreditation bodies for IPE. No accreditation body for IPE still exists.

## Discussion

We reviewed 40 published articles on implementation of IPE. Out of 40, 38 articles were identified from developed countries. Then, we identified ten important challenges and barriers while planning, initiating, and implementing IPE. They included IPE curriculum, leadership, resources, stereotypes and attitudes, variety of students, IPE concept, teaching, enthusiasm, professional jargons, and accreditation. Implementation of IPE programs in developed countries was possible despite the challenges described. When we compared these ten challenges and barriers with two studies from developing countries, we found they identified only three out of the 10 challenges identified in studies from developed countries. It does not mean that the remaining seven were not considered to be challenges or barriers. When IPE programs are increasingly implemented in developing countries, they may face all of them, and it is critical to know these potential challenges in advance.

The studies from developed countries provide examples of implementation of IPE courses despite the above challenges and barriers. These lessons from the past will suggest a way forward for IPE implementation in developing countries.

### Anticipated challenges in implementing IPE in developing countries

Evidence is scarce on IPE conduct in developing countries. In this review, only two articles were identified from such countries [25], [26]. IPE curriculum structure and complexity, resource limitations, and stereotypes were the three challenges identified from these articles. This evidence may not be generalized to the rest of the countries, as the number is limited. However, examples of challenges and barriers encountered from these and from developed countries can help guide planning, initiating, and implementing IPE in other developing countries.

Money and workforce limitations were barriers in developing countries [26]. Sustainability in funding is a common problem in most medical training institutions in developing countries [63]. The majority of public-owned and operated institutions rely on low budgets from governments [63]. They face difficulties in running even their basic professional courses [64]. Adding a new IPE course based on the same level of budget will impede its implementation and sustainability. Similar approaches to those of developed countries could be considered in the context of developing countries. For example, integrating IPE into the existing curriculum can save the added cost of running parallel curricula [25]. Existing faculties can be involved in its design, initiation, and implementation with minimal or no added costs. Students would not need to travel to other campuses for IPE if faculties within the same university implement IPE together. Seeking independent funds and partnerships with global initiatives can expand the sources of available funds [65].

In developing countries, even faculty members for basic and clinical sciences in training institutes are scarce and those available are underpaid [63], [66]. This can impede planning, initiating, and implementing IPE programs. In most of the IPE models in developed countries, a separate IPE course was established with a separate set of faculty members. Such a model is not sustainable in areas with inadequate staff to carry out even the existing professional courses [64]. In developing countries, also, highly qualified and specialized medical professionals are not adequate to guide students through clinical practice. The available specialists are responsible for a high burden of patients [64]. They may not have the time or motivation to teach large IPE classes on clinical training. In this review, some IPE programs that faced such problems in developed countries integrated IPE into the existing professional courses. Available staff received training to help them teach students from different professional backgrounds [67]. Smaller classes were made to foster interactions and programs used the existing teaching hospitals at their training institutions [42]. Similar approaches may bear the desired fruits if adopted by developing countries [25].

The limited number of medical training institutions [63] and large number of students are another common problem in developing countries. IPE has been tested in developed countries with a large number of medical training institutions that have classes with small numbers of students. An IPE class with a small number of students can enhance interactions among different professions and faculties [13]. Clinical practice, especially in PBL, is also efficient in small groups [68]. However, similar approaches might not be possible in developing countries. Models should be built by each institution based on their own situation [69]. Dividing classes into smaller groups [70] can be one approach but might be possible only if the institution has enough physical space and faculty members to manage several groups [13].

Stereotypes, negative attitudes, and the role of professional bodies should not be overlooked in developing countries [25]. Medical doctors in developing countries also tend to be powerful, as are their medical students relative to students in other professional programs. Medical doctors and students tend to be leaders and others act as team players. This attitude is against the spirit of interprofessional collaboration [9], [71]. IPE planners should take this into consideration and conduct pre-session training to foster team building and collaboration before the IPE course [72]. Professional bodies such as medical associations may not be strong in developing countries but should also be involved in planning and implementing IPE. Their members are faithful to these organizations. Changing the mindset of senior professionals can be an important approach to mitigating the effects of stereotyping on IPE.

Lack of accreditation of IPE was a barrier in studies conducted in developed countries [73]. In developing countries, introduction of IPE will also face a similar challenge. Lack of accreditation will make such courses less standardized and serious [74]. In most developing countries, even accreditation for regular medical schools remains a problem. Different medical schools may have different styles and approaches to delivering their courses. It may thus be difficult to synchronize IPE among medical schools with different approaches towards similar goals. Efforts to standardize medical schools are necessary and will provide a favorable platform for IPE in such countries, bolstered by accreditation of IPE courses. However, it remains debatable if accreditation of medical schools must be done as a prerequisite for IPE programs.

Poor leadership was a barrier to implementation of IPE in developed countries. For this, evidence is also available from developing countries but it is limited and remains a challenge. In developed countries, strong leaders and their commitment towards IPE were an important reason for success [75]. Similar programs should take advantage of people interested in IPE as champions for the programs in developing countries. School administrators and deans should be involved from the planning stage. Students and other faculties should also take the lead in planning and implementing IPE. In addition, involving the administrators might convince them to provide sufficient budget to help implement and sustain IPE programs.

### Tailored approach can help design a sustainable IPE program in developing countries

In developing countries, an IPE program should be designed based on the context of the area in which it is to be implemented. Key stakeholders should be involved throughout the process of planning, initiating and implementing the IPE program. Schedules and timetables should also be taken into consideration. The schedules of professional schools are tight, faculty members are limited, and the number of students tends to be very large. IPE should take advantage of the case studies that successfully generated interest among the participants, and most importantly, aim to solve health problems pertinent to the area [66], [76]. Interactions among professionals may be accompanied by some conflict. Such challenges should be solved promptly and amicably. Evaluations by students, faculties, and patients in clinical learning should be conducted frequently and taken seriously for IPE program sustainability.

IPE is important for developing countries despite the likely challenges and barriers. Indeed, they should be taken as opportunities to solve the core health problems in developing countries. For example, IPE can help to foster interprofessional collaboration (IPC) and to address the problem of resource constraints [2]. With strong IPC, a large number of patients in developing countries can be shared within a team and responsibilities can be shifted among cadres of different professional backgrounds. Lack of continuing medical education (CME) and continuing professional development (CPD) in developing countries can partly be solved through IPC resulting from IPE. For example, in well-established settings where health personnel work as a team, senior and experienced health workers can share their knowledge among other staff including those more junior and less experienced. As in developed countries [2]–[6], IPE in developing countries can improve professionals' medical and clinical knowledge, skills, and professional practices [7]. This will also boost patient satisfaction [7].

### Study limitations

The results of this study should be interpreted in light of two limitations. First, we did not find adequate evidence from developing countries where we aimed to make our suggestions. However, the challenges and barriers faced by IPE in developed countries are likely relevant to developing countries where resources are even scarcer. Second, we could not conduct a meta-analysis due to the wide differences in study design, populations, settings, and presented results. However, the meta-narrative is more explanatory because challenges and barriers are best measured qualitatively.

### Conclusion

This review found a number of challenges and barriers to IPE implementation in studies conducted in developed countries. They are: curriculum, leadership, resources, stereotypes and attitudes, variety of students, IPE concept, teaching, enthusiasm, professional jargons, and accreditation. Out of these 10, three were described in studies conducted in developing countries: resource limitation, leadership challenges and stereotypes. However, challenges observed in this review suggest that the remaining seven are also potentially important for developing countries. By being aware of these challenges and barriers in advance, those who seek to plan and implement IPE programs in developing countries will be much more prepared and their efforts may proceed more smoothly and successfully.

## Supporting Information

Checklist S1 PRISMA Checklist.
(DOC)
